# Making Waves: Biocatalysis and Biosorption: Opportunities and Challenges Associated with a New Protein-Based Toolbox for Water and Wastewater Treatment

**DOI:** 10.1016/j.wroa.2021.100112

**Published:** 2021-08-03

**Authors:** Justin M. Hutchison, Brooke K. Mayer, Marcela Vega, Wambura E. Chacha, Julie L. Zilles

**Affiliations:** a Department of Civil, Environmental, and Architectural Engineering, University of Kansas, 1530 W 15th St, Lawrence, KS 66045, United States; b Department of Civil, Construction and Environmental Engineering, Marquette University, 1637 W Wisconsin Ave., Milwaukee, WI 53233, United States; c Department of Crop Sciences, University of Illinois at Urbana-Champaign, 1102 S Goodwin Ave, Urbana, IL 61801, United States

**Keywords:** Biocatalysis, Biosorption, Perchlorate, Perchlorate reductase, Phosphate-binding protein

## Abstract

New water and wastewater treatment technologies are required to meet the demands created by emerging contaminants and resource recovery needs, yet technology development is a slow and uncertain process. Through evolution, nature has developed highly selective and fast-acting proteins that could help address these issues, but research and application have been limited, often due to assumptions about stability and economic feasibility. Here we highlight the potential advantages of cell-free, protein-based water and wastewater treatment processes (biocatalysis and biosorption), evaluate existing information about their economic feasibility, consider when a protein-based treatment process might be advantageous, and highlight key research needs.

## Highlights

• Protein-based approaches are promising for sustainable water/wastewater treatment.
• Biocatalysts can convert low concentration contaminants to nontoxic end products.
• Biosorbents can selectively remove contaminants and recover resources from water.

## Graphical abstract

**Figure:**
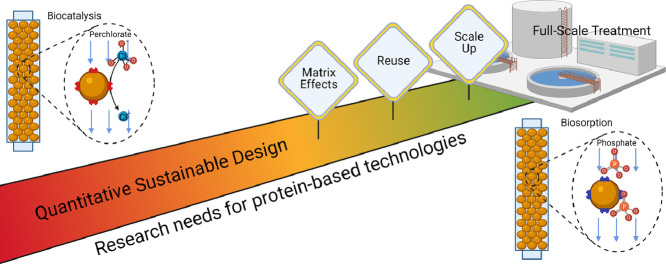

As we learn more about the environmental distribution and fate of chemicals and their effects on human and environmental health, facilities treating water are increasingly expected to remove a broad range of contaminants and achieve ever higher water quality targets. Furthermore, some of these contaminants have intrinsic value, if they could be efficiently recovered in forms suitable for reuse, e.g., nutrients for agricultural fertilizer or precious metals and rare earth elements as industrial components. At the same time, due to anthropogenic impacts on climate, treatment processes are also under pressure to reduce their energy requirements and environmental impacts. These are urgent, important, and complicated drivers; these issues cannot be resolved by a single solution. Part of the solution lies in expanding source control, while other parts depend on using more holistic, broader criteria to design treatment systems and developing a larger set of processes to draw on. Biology holds a powerful set of tools that have great potential in water treatment: proteins that can catalyze reactions (enzymes or biocatalysts), sorb specific compounds (non-enzymatic biosorbents), or transport specific compounds (transporters). This perspective piece focuses on the potential utility, limitations, and research needs of biocatalysis and biosorption for drinking water and wastewater treatment applications. For both biocatalysis and biosorption, our definition is restricted to cell-free proteins, not encompassing any whole cell applications.

This biological toolbox exhibits several potentially beneficial characteristics. First, in comparison to chemical catalysts, proteins generally function best under mild conditions typical of water and wastewater treatment. Biocatalysts can maintain optimal activity over a pH range from 6.5 to 9 and exhibit a predictable activity temperature dependence (Hutchison and Zilles, 2015). Similarly, biosorbents can bind substrates with minimal decrease in efficiency between pH 4.7 and 8.5 (Venkiteshwaran et al., 2018). For treatment configurations (e.g., enhanced coagulation, optimal corrosion control, precipitative softening) that might fall outside the operational range of specific biocatalysts and biosorbents, protein-based technologies could likely be placed elsewhere in the treatment train.

Second, proteins often have high affinity for their substrate(s), which is an advantage when treating micropollutants and other contaminants characterized by increasingly low regulatory limits (e.g., phosphorus). Third, biocatalysts have the potential for complete degradation of contaminants (e.g. (Hutchison and Zilles, 2018)), avoiding problems associated with transformation products. Fourth, both biocatalysts and biosorbents vary in their degree of specificity, which provides an opportunity to tailor process design. For example, more promiscuous biocatalysts such as laccase have been proposed to treat a wide range of contaminants (Chen et al., 2016; Garcia et al., 2011; Lloret et al., 2013). On the other end of the specificity spectrum, the high specificity of perchlorate reductase is advantageous because it allows removal of perchlorate in the presence of much higher concentrations of co-contaminating nitrate (Hutchison et al., 2013), a situation that ion exchange and whole-cell biological processes have difficulty with. Moreover, selective biosorption processes can enable recovery of the target without contamination from other constituents (e.g. (Venkiteshwaran et al., 2018, 2020)). An ideal biosorption process would leverage highly selective, sensitive, and reversible (under controlled conditions) biosorption to facilitate recovery in a concentrated, contaminant-free form suitable for reuse.

While cell-free biocatalysis is widely applied in pharmaceutical and industrial biotechnology processes, it has not yet been widely implemented for drinking water or wastewater treatment applications. The high volume and low profit margin of water treatment industries present challenges not faced in the pharmaceutical industry, contributing to the most frequently voiced concern: that biocatalysts are too expensive. To our knowledge, protein biosorbents are not yet in use industrially or in water treatment, although they have been proposed for metals (Han et al., 2017; Keshav et al., 2019; Maruyama et al., 2007; Xiao et al., 2020) and phosphorus removal and recovery (Venkiteshwaran et al., 2018, 2020). In this article, we examine the state of knowledge concerning the economic feasibility of biocatalysis and biosorption for drinking water and wastewater treatment. We then consider what types of applications might be most amenable to biocatalysis or biosorption and conclude by highlighting key research needs.

## Evaluating the economic feasibility of biocatalysis and biosorption

We begin with the specific case of biocatalysis for perchlorate removal from drinking water, because this has been studied in more detail. Our recent technoeconomic assessment suggested that biocatalysis could be a cost-competitive treatment for perchlorate (Hutchison et al., 2017). Specifically, with directed research investment, biocatalytic perchlorate drinking water treatment was projected to cost $0.034 *m*^−3^, with global warming potential (GWP) impacts of 0.051 kg CO_2_ eq *m*^−3^. These values compare favorably to overall water treatment costs and environmental impacts, constituting a potential increase of 6.5% in costs and 7.3% in GWP impacts compared to total drinking water treatment plant operations without perchlorate removal. In our analysis, these costs and impacts were also comparable to alternative technologies at a more advanced stage of development: idealized biological perchlorate reduction and perchlorate-selective ion exchange.

These results are quite promising for an early-stage technology; why are they so different from common expectations? Perceptions that biocatalysis and biosorption are too expensive often derive from concerns about protein stability, effects of complex water matrices on proteins, and production costs. Data from perchlorate-reducing enzymes demonstrate that these concerns are not universally applicable. First, perchlorate-reducing enzymes are quite stable, maintaining 58.2 % of initial activity up to 23 days, without any effort to optimize stability (Hutchison et al., 2013). A variety of approaches are also available to enhance protein stability, including genetic engineering (Chandler et al., 2020) and encapsulation in materials such as gels (Zhang et al., 2015) or vault nanoparticles (Wang et al., 2015). Second, perchlorate-reducing enzymes maintain high activity in real drinking water sources (Hutchison and Zilles, 2015) and ion exchange regeneration brines (Hutchison and Zilles, 2018). The third concern, production costs, is more difficult to evaluate, since existing production protocols have been developed for research purposes and are not optimized for large-scale production. However, models for scale-up are available from the pharmaceutical industry, and experience curves from a wide range of industries show that substantial reductions in cost are typically achieved during technology scale-up and development (Thomassen et al., 2020; Wene, 2000). These results illustrate the potential of this new toolbox; although key technology developments are needed, economic feasibility appears attainable.

Implementing biocatalysis in a way that allows reuse of the proteins is essential for cost-effective perchlorate treatment (Hutchison et al., 2017), and likely for biocatalytic and biosorption approaches in general, as others have noted previously (e.g. (Sheldon, 2007; Tufvesson et al., 2010; Zhu et al., 2019)). A wide variety of approaches are available for protein reuse (e.g. (Cao, 2005; Minteer, 2011)). Several of these approaches have been investigated for environmental applications, including immobilizing proteins for use in fixed bed, regenerable, ion exchange-style systems (e.g. (Venkiteshwaran et al., 2018)); displaying them on a cell surface (e.g. (Chen et al., 2016; Hussein et al., 2020; Zhu et al., 2019)); or attaching them to beads or nanotubes which can be settled, filtered out, or recovered with a magnetic field, depending on their size (e.g. (Kumar and Cabana, 2016; Zhu et al., 2021)). Despite decades of research in the pharmaceutical industry, identifying an immobilization strategy that maintains or enhances activity and stability for a given protein remains largely a matter of trial and error. Integration of data-driven approaches such as materials informatics (Jones et al., 2020) has the potential to accelerate design of new materials featuring effective immobilization, as discussed in a recent review (Zhu et al., 2019). For our purposes here, the important point is that immobilization and reuse are technically feasible.

To our knowledge, robust TEA and LCA analyses for contaminant removal and/or recovery from water or wastewater using cell-free, protein-based biosorbents have not been conducted. However, two TEAs focused on the use of intact bacterial cells for sorption and recovery of rare earth elements from industrial byproducts identified specific applications that were economically viable (Alipanah et al., 2020; Jin et al., 2017). Both studies incorporated immobilization and reuse of the whole-cell biosorbents, which was critical for economic and environmental performance. Considering stability and matrix complexity, the surface-expressed peptide tags on these cells were active in a range of complex matrices, including acid leachate and geothermal brine (Jin et al., 2017).

For biosorption, in addition to the concept of sorbent reuse, new dimensions come into play. Biosorbents not only need to be retained or recovered for reuse; their sorption capacity also needs to be regenerated under controlled conditions, to release the target sorbate (analogous to reversible, selective inorganic ion exchange). Like reuse, regeneration is technically feasible. For example, phosphorus-binding capacity was sustained over ten cycles of neutral and high pH washes that promoted adsorption and desorption, respectively (Venkiteshwaran et al., 2018). From a cost perspective, biosorption has additional potential benefits; recovery of the compound being removed in a concentrated (high affinity), pure form (high specificity) could advance the waste valorization paradigm.

Other considerations may be universal or specific to certain biocatalysts or biosorbents. As with any emerging technology, infrastructure buildup is necessary to supply protein-based materials. The protein production scale required for the technologies proposed here is an ongoing field of study. However, large-scale applications are being pursued commercially for biocatalytic recycling of polyethylene terephthalate plastics (Albert, 2020).

Although stability and sensitivity to complex water matrices were not problematic for perchlorate biocatalysis, these important characteristics affect the economic feasibility of biocatalysis and biosorption and will likely vary across proteins. However, even in cases where stability and/or sensitivity are problematic, there are established routes for improving protein characteristics, including mining biological diversity (Mobilia et al., 2017), applying directed evolution (Arnold, 1996), and using structure-based approaches (Sammond et al., 2007). On the whole, then, realizing these technology improvements could put the economic feasibility of biocatalysis and biosorption for water and wastewater treatment within reach.

For perchlorate reduction, which requires reducing power, another major technology development goal required for sustainable application is identifying effective and scalable means of supplying electron donors (Hutchison et al., 2017). Identifying suitable sources of electron donors or acceptors will be important for redox reactions, while other types of reactions might require specific cofactors or cosubstrates, and yet others might not require anything besides the biocatalyst and the contaminant (Mobilia et al., 2017). For biosorbents, the materials needed for regeneration will be a key consideration.

## Determining when biocatalysis or biosorption might be appropriate

**Fig. 1:**
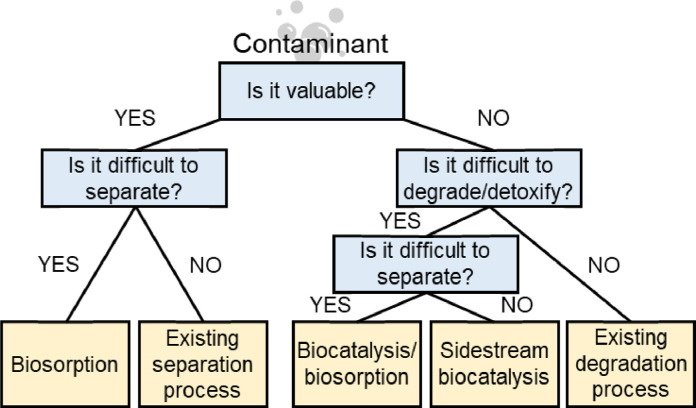
Biocatalyst and Biosorption Decision Tree: To identify applications where the long-term prospects of biosorption and biocatalytic technologies are promising, the context of existing technologies and markets is important.

A decision to investigate one of these approaches should be based on specific characteristics of the contaminant(s). In general, contaminants with recovery value are good candidates for biosorption, while contaminants that are difficult to treat with existing methods are good candidates for biocatalysis (Fig. 1). Biosorption could assist with the recovery of economically-valuable constituents in water, facilitating the shift from wastewater treatment plants to water resource recovery facilities. For contaminants that have little to no economic value, transformation into innocuous end products is beneficial. Classification of a compound as a contaminant generally depends on it having biological effects. Since cells evolve over time to take up useful resources and to expel or degrade toxic compounds, potential biosorbents or biocatalysts are likely to be naturally occurring for many contaminants. In cases where current technologies are unable to realize the full potential of resource recovery or contaminant degradation, then, existing proteins may provide new treatments based on biosorption/biocatalysis.

Biocatalysis and biosorption can be applied on their own or within hybrid processes. Hybrid processes in which biocatalysis or biosorption play a supporting role may provide particularly good opportunities to rigorously evaluate performance and reliability in water and wastewater treatment and to develop operational experience with these emerging technologies. For example, sidestream biocatalysis could be used to regenerate spent anion exchange brine (Hutchison and Zilles 2018), enhancing the sustainability of ion exchange processes (Lehman et al., 2008). Phosphate-binding proteins could facilitate anaerobic digestion of enhanced biological phosphate removal (EBPR) biosolids by capturing the released phosphate and allowing its recovery.

Specifically comparing biocatalysis and biosorption to biological processes, in general whole cell biological processes should be investigated first. They are likely to be less expensive, because the cells are biologically regenerated within the process. However, there are a variety of applications where whole cell processes are ineffective or uncompetitive. For example, when a contaminant is present at low concentrations or in sporadic pulses, it might be unable to sustain a whole-cell process but could be amenable to treatment using cell-free proteins. In other cases, there might be health risks, regulatory restrictions, and/or public relations concerns associated with the use of whole cells, such as in drinking water treatment in the United States. While the health risks associated with any specific biocatalyst or biosorbent need to be evaluated, the building blocks of proteins are nontoxic, and their inability to reproduce reduces the risk of widespread contamination, as compared with whole cells. Biocatalysis and biosorption can also provide more opportunities for a targeted process. For example, biocatalysis allows removal of perchlorate in the presence of excess co-contaminating nitrate (Hutchison et al., 2013), a condition where whole cells preferentially remove the nitrate. Likewise, phosphorus-binding proteins are being investigated as biosorbents, in part because of the potential to selectively recover phosphorus, even in the presence of structurally similar oxyanions such as sulfate and arsenate, which can co-adsorb to inorganic adsorbents (Venkiteshwaran et al., 2020, 2021). Even when compound recovery may not be a priority, biosorbents’ selectivity and sensitivity may still have utility, such as with ultra-low treatment targets (e.g., 10 µg/L arsenic).

**Fig. 2:**
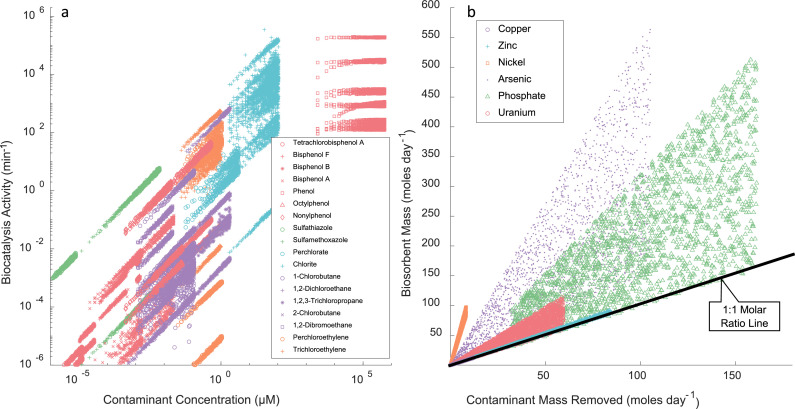
Biocatalytic and Biosorption Treatment Landscapes. a) Biocatalytic activity was calculated using the Michaelis-Menten kinetics equations, published kinetic parameters of V_max_ and K_m_, and environmentally relevant contaminant concentrations. b) Biosorption was evaluated based on the amount of biosorbent required to remove the contaminants at a flow rate of 1 million liters per day. This amount was calculated using environmentally relevant contaminant concentrations, established regulatory limits, protein-specific dissociation constants (K_d_), and a Langmuir isotherm. Contaminants were selected based on the availability of enzyme kinetic information (biocatalysis) or binding affinity (biosorption) and their classification as contaminants of emerging concern, the presence of a U.S. EPA regulation, and/or their economic value. Environmentally relevant concentration ranges were developed based on published measurements from surface and groundwaters or wastewater influent. The low end of the contaminant range was further refined based on published health advisory limits available in the literature. For both graphs, to account for the data uncertainty (e.g., standard deviations), Monte Carlo simulations (200 for biocatalysts and 2000 for biosorbents) were performed with Latin Hyper Cube Sampling. In cases where standard deviations were not available, a uniform distribution +/- 10% of the reported values was used. The full datasets and associated references can be found in the Mendeley data repository http://dx.doi.org/10.17632/wt4mm84xv2.1. Code used to analyze data and create figures is available at https://github.com/jhutchku/2021_03_MakingWaves.git.

To investigate the potential treatment landscape for biocatalysis and biosorption in more detail, we collected and modeled key protein characteristics and compared them to environmental contaminant concentrations. Specifically, for biocatalysts, kinetic parameters for activity (V_max_) and substrate affinity (K_m_) were used in conjunction with the Michaelis-Menten equation to calculate enzyme activity (µmole min^−1^ µmole^−1^ simplified to min^−1^) for contaminant concentration ranges typical of environmental waters and wastewaters. The resulting contaminant concentrations and corresponding biocatalyst activities ranged across 12 orders of magnitude, a large treatment landscape (Fig. 2a). Typically, biocatalyst activity profiles would have a characteristic plateau at maximum activity, as seen with the phenol degrading biocatalysts. This plateau is missing for several enzymes because the environmentally relevant contaminant concentrations used here were too low to support maximum activity. For biocatalysts on the lower part of the graph, where activity is relatively low at relevant concentrations, optimization of substrate affinity might be necessary. This research need has been noted previously, for example in a study using laccase to remove tetracycline (Abejón et al., 2015). Nonetheless, biocatalysts provide advantages over chemical catalysts, including using earth-abundant, nontoxic metals in lieu of the rare and hazardous metals common to traditional chemical catalysts and having faster kinetics. For example, the perchlorate-reducing biocatalysts’ activity value is 41,000 times greater than rhenium-palladium-based perchlorate-reducing catalysts (Hutchison and Zilles, 2015). Even more noteworthy are the extreme reaction potentials achieved by the fastest biocatalysts, which can push the upper bounds of the activity graph to ∼10^9^ min^−1^ (Ogura and Yamazaki, 1983).

For biosorbents, the relevant parameter is the equilibrium dissociation constant (K_d_), which is a measure of the protein's binding affinity for the contaminant. The inverse of K_d_ is equivalent to the Langmuir equilibrium constant. To calculate the results in Fig. 2b, the Langmuir isotherm was integrated in a continuously-stirred tank reactor mass balance with a flow rate of one million liters per day. A uniform distribution of environmentally-relevant contaminant concentrations was used to determine the moles of biosorbent required to achieve effluent targets. Some precious metals were excluded from the figure due to their low environmental concentrations. Several of the phosphate-binding proteins are approaching an ideal binding efficiency frontier of 1:1 molar ratio of biosorbent to contaminant over a range of contaminant removals. For these proteins, further efforts to optimize binding efficiency (or to decrease the K_d_ value) may not yield substantial improvements. In comparison, the arsenic binding protein modeled here shows room for improvement in binding efficiency.

## Key research needs

As detailed in this perspective piece, the available data suggest that biocatalysis and biosorption could be economically viable for particular water and wastewater applications. Our comparison of kinetic parameters (for biocatalysts) and dissociation constants (indicative of binding strength for biosorbents) with environmentally relevant contaminant concentrations suggests the potential treatment landscape is large. We turn therefore to highlighting key research needs for development of this promising new water treatment toolbox.

As detailed above, development and demonstration of repeated cycles of reuse is a key technology development goal. Although a few studies have demonstrated cycles of reuse (e.g. (Chen et al., 2016; Venkiteshwaran et al., 2018)), little data is available to show how many cycles might be feasible, particularly in natural water and wastewater matrices, and support materials have not been optimized for these applications. More generally, the many different approaches for immobilization and/or recovery likely vary in their associated trade-offs, including effects on activity, mass transfer limitations, and retention/recovery efficiency. Strategic use of integrated life cycle analysis (LCA) and technoeconomic assessment (TEA) that include potential technology improvements, also referred to as prospective assessments or quantitative sustainable design (Shi and Guest, 2020; Thomassen et al., 2019), should continue to guide technology development (Hutchison et al., 2017), establishing a robust framework for development of biocatalytic and biosorptive treatment processes. It would be particularly helpful to have a representative LCA/TEA for biosorption, as the value of recovered product could shift the economics further into the competitive realm.

## Conclusions

• Biocatalysis and biosorption have the potential to provide robust, high activity/capacity, tailored specificity, and low-cost processes, improving our ability to meet water and wastewater treatment goals. Biosorption additionally could facilitate resource recovery. This is a research area ripe for advancement.
• Key research needs include i) evaluating different approaches for biocatalyst/biosorbent reuse and their associated impacts on activity, stability, mass transfer, and flow rates and ii) conducting TEAs and LCAs, to guide technology development toward the most problematic issues and the most promising implementations and applications.

## Declaration of Competing Interest

The authors declare that they have no known competing financial interests or personal relationships that could have appeared to influence the work reported in this paper.
